# USP14 maintains HIF1-α stabilization via its deubiquitination activity in hepatocellular carcinoma

**DOI:** 10.1038/s41419-021-04089-6

**Published:** 2021-08-21

**Authors:** Chi Lv, Shengli Wang, Lin Lin, Chunyu Wang, Kai Zeng, Yiming Meng, Ge Sun, Shan Wei, Yefu Liu, Yue Zhao

**Affiliations:** 1 Department of Cell Biology, Key laboratory of Cell Biology, Ministry of Public Health, and Key laboratory of Medical Cell Biology, Ministry of Education, School of Life Sciences, China Medical University, Shenyang City, Liaoning Province China; 2 Department of General Surgery, Cancer hospital of China Medical University, Liaoning Province Cancer Hospital, Shenyang, Liaoning China; 3 Anorectal Surgery Ward, Department of General Surgery, Shengjing Hospital of China Medical University, Shenyang City, Liaoning Province China

**Keywords:** Cancer, Cell biology

## Abstract

Hepatocellular carcinoma (HCC) is the most common visceral neoplasms with its heterogeneity and high rate of recurrence. HCC is characterized to be delayed diagnosis and the development of resistant disease. However, the molecular mechanism for HCC pathogenesis and progression remains largely unknown. Here, we demonstrated that ubiquitin-specific protease14 (USP14) is highly expressed in HCC samples, and the higher expression of USP14 is positively correlated with poor prognosis. Interestingly, USP14 is involved in the maintenance of HIF1-α stability to activate HIF1-α-induced transactivation via its deubiquitinase activity. USP14 depletion or its specific inhibitor IU1 treatment decreased cell proliferation, invasion, migration, and Vascular Mimicry (VM) formation even under hypoxia conditions in HCC cell lines. Moreover, we provided the evidence to show that knockdown of USP14 or USP14 inhibitor (IU1) treatment inhibited tumor growth in tumor-bearing nude mice. Our findings suggest that USP14 maintains HIF1-α stability through its deubiquitination activity, providing a potential biomarker for the early diagnosis and therapy of HCC.

## Introduction

Hepatocellular carcinoma (HCC) is one of the most serious carcinomas, with a 5-year survival rate is 14–18%. HCC is the third cause of cancer mortality worldwide [1]. Despite the emergence of promising treatment strategies, most of the patients had advanced to the middle and late stages due to the difficulty in early diagnosis for HCC [2]. Even with surgical treatment, the recurrence rate within 5 years was as high as 40–60% [3]. Therefore, it is urgent to gain insight into the molecular mechanism of tumorigenesis and progression of HCC to explore potential therapeutic targets for HCC patients.

Hypoxia is a hallmark for solid tumors microenvironment, which is attributed to the vasculature reformation and metabolic reprogramming [4]. Hypoxia-induced factor (HIF-1) is the major regulator of oxygen homeostasis. HIF-1 consists of two subunits: one is oxygen-sensitive HIF-1α subunit, assisting the cellular adaptive response to hypoxia, the other is the constitutively expressed HIF-1β subunit [5]. HIF-1α as a transcriptional regulator is recruited to the hypoxia response element (HRE) of target genes to induce the transcription of a series of target genes, including VEGF, MMP2, MET, TWIST and so on [6]. The previous study has shown that HIF-1α-mediated genes give rise to angiogenesis and metastasis, which are crucial for the development and progression of HCC [7].

HIF1-α degradation has been reported to be triggered by ubiquitination via a von Hippel–Lindau (VHL)-containing E3 ubiquitin ligase [8]. It has been mentioned that some proteins involved in the maintenance of HIF1-α stability play crucial roles in tumorigenesis and progression of HCC. Ubiquitin-specific protease 22 (USP22) stabilizes HIF1-α to promote hypoxia-mediated HCC stemness and glycolysis by its deubiquitinase activity [9]. CDC20 as a cell cycle regulator mediates PHD3 degradation to increase the stability of HIF1-α in HCC [10]. Thus, understanding well the molecular mechanism underlying the modulation of HIF1-α stability would be essential for finding the potential therapeutic targets for HCC.

Ubiquitin-specific protease 14 (USP14) as a member of the Ubiquitin-specific proteases (USP) protein family interacts with the 26 S proteasome complex [11] and enhances its deubiquitination by reversibly binding to the Rpn1 in the proteasome 19S regulating particle [12]. USP proteins are the antagonists of E3 ligases to be increasingly recognized as potential targets in cancer treatment [13]. IU1 is one of the selective small-molecule inhibitors of deubiquitination activity of USP14, and IU1 is able to accelerate proteolysis in cells [14]. It has been demonstrated that a specific USP14 and UCH37 inhibitor b-AP15 inhibits the growth of tumor with *P53* deficiency [15]. Moreover, inhibition of USP14 increased AR degradation and suppressed the AR-mediated signalling pathway to enhance the sensitivity of AR-positive breast cancer to AR antagonist (enzalutamide) [16]. USP14 plays an indispensable role in hepatosteatosis through the stabilization of FASN [17]. However, the function of USP14 on HCC progression is still elusive.

In this study, our results have shown that USP14 participates in the maintenance of HIF1-α stability and enhances HIF1-α-induced transcriptional activity via its deubiquitination activity in HCC cell lines. In addition, USP14 is highly expressed in clinical HCC samples, and higher expression of USP14 is positively correlated with the poor prognosis in HCC. USP14 depletion or USP14 specific inhibitor IU1 significantly suppresses cell growth, migration, and VM formation in HCC. Moreover, we provided the evidence to demonstrate that knockdown of USP14 or IU1 treatment inhibited tumor growth in mice. Our findings indicate that USP14 as a deubiquitinase is involved in maintaining HIF1-α stability and enhancement of HIF1-α activity, suggesting USP14 would be a potential diagnosis biomarker and therapeutic target for HCC.

## Results

### USP14 is highly expressed in HCC samples, and the higher expression of USP14 is positively correlated with poor prognosis

**Fig. 1:**
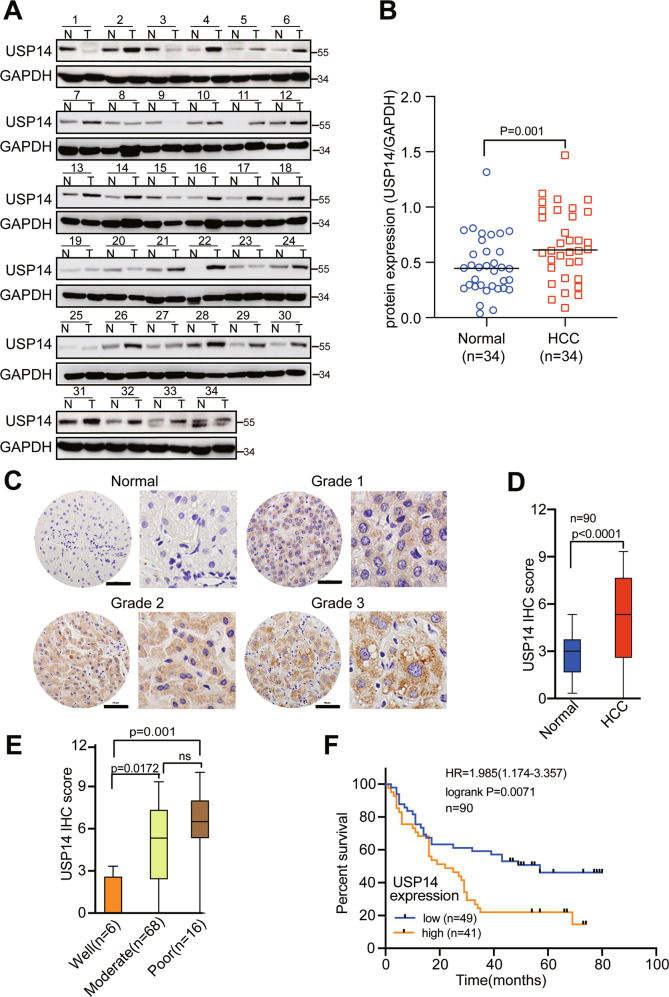
Upregulation of USP14 predicts poor clinical outcome in patients with HCC. **A** The protein expression of USP14 in HCC tissues were examined by western blot. N stands for the adjacent non-tumorous tissues, T stands for HCC tissues. **B** The representative images and the statistical analysis were indicated. **C**, **D** The expression of USP14 in adjacent non-tumorous tissues, HCC tissues with different levels of differentiation was detected by IHC. Scale bar, 100 μM. **E** The representative images and the IHC scores were shown. **F** The clinical significance of USP14 expression in overall survival was evaluated in the TMA-based cohort by Kaplan–Meier survival analyses.

We first analyzed The Cancer Genome Atlas (TCGA) data using the UALCAN online tool (http://ualcan.path.uab.edu/index.html). The results showed that USP14 mRNA expression was significantly higher in primary HCC tissues than that in normal liver tissues (Figure S1A). Additionally, USP14 protein is highly expressed in HCC specimens compared with that in normal liver samples according to The Human Protein Atlas (http://proteinatlas.org/), which is a public repository of immunohistochemistry data (Figure S1B). UALCAN online tool analysis demonstrated a significant correlation between USP14 mRNA expression and clinical cancer stages and histological grades (Figure S1C, D). Moreover, an online Kaplan–Meier Plotter analysis tool (http://kmplot.com) showed that HCC patients with high USP14 expression had significantly decreased overall survival and relapse-free survival (Figure S1E, F). Consistent with the above-mentioned bioinformatics online database analysis, western blotting data demonstrated that USP14 protein was highly expressed in 34 pairs of fresh clinical HCC samples (T) compared with that in the adjacent non-tumor tissues (N) (Fig. 1A, B). Immunohistochemistry (IHC) results containing 90 pairs of HCC samples demonstrated that USP14 was highly expressed in HCC tissues compared with those in the adjacent tissues (Fig. 1C, D).

The HCC patients were divided into the high USP14 and low USP14 groups according to the IHC score. The median IHC score of 6.0 was chosen as the cut-off value to define the high and low expression of USP14. Our results showed that high USP14 expression was positively associated with the differentiation of HCC, but has no relationship with other clinicopathological features, including age, gender, cirrhosis, tumor size, perihepatic organ invasion, vascular invasion, lymph node metastasis, and TNM (tumor, lymph node, and metastasis) state (Fig. 1E, Table S1). The prognostic value of USP14 was further explored. Our results showed that highly expression of USP14 was positively correlated with the poor prognosis (*p* = 0.0071, HR = 1.985) (Fig. 1F). The median overall survivals in high USP14 and low USP14 groups were 22 and 44 months, respectively. Multivariate analyses using the Cox regression model revealed USP14 as an independent prognostic factor for overall survival in HCC (Table S2). Collectively, these data suggest that USP14 is highly expressed in HCC samples, and higher expression of USP14 is positively correlated with malignant progression and the poor prognosis in HCC.

### USP14 enhances HIF1-α-mediated transactivation in HCC cells

**Fig. 2:**
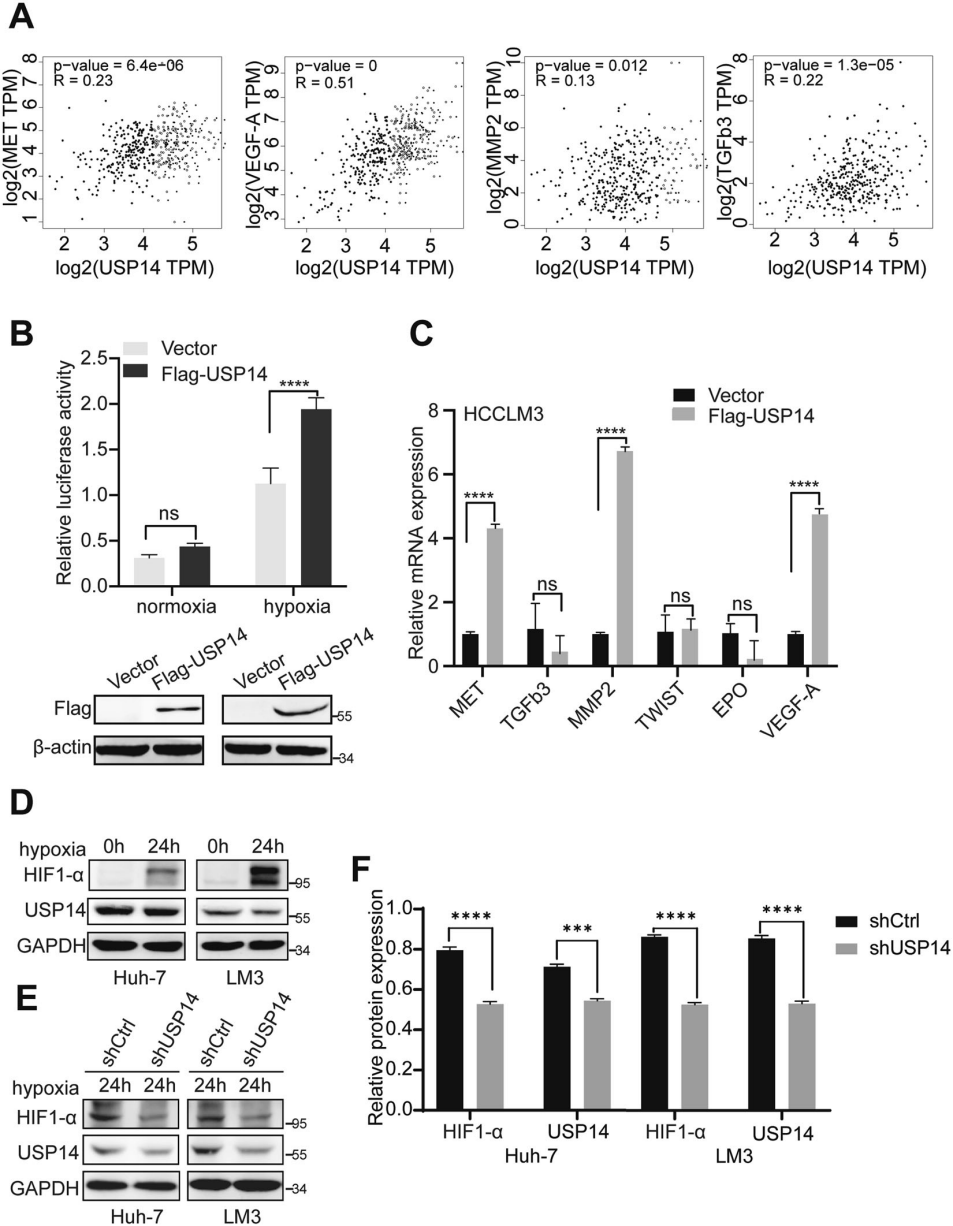
USP14 enhances HIF1-α-mediated transactivation in HCC cells. **A** Studies from the GEPIA database presented the correlation between USP14 mRNA expression and HIF1-α target genes’ mRNA. **B** Fold change in the relative luciferase activity was examined by luciferase reporter assay in USP14 overexpression or vector HCCLM3 cells incubated under normoxia or hypoxia (in 1% O2 incubator) for 24 h (up panel). Cell lysates from the luciferase reporter assay were collected and analyzed simultaneously by immunoblotting (down panel). **C** Real-time PCR analysis showed the effect of USP14 overexpression on activation of HIF1-α target genes in hypoxia. **D** HIF1-α protein increased in two HCC cells under hypoxia state induced by CoCl_2_. **E** The protein expression levels of HIF1-α in control and USP14 knockdown HCC cells were examined by western blot. **F** Quantification of protein expression is shown as histograms on the right. In the histogram, the bars represent the mean ± SD (*n* = 3), *P < 0.05, **P < 0.01, ***P < 0.001.

In order to explore the mechanism underlying the function of USP14 in HCC progression, we turned to analyze the data from the GEPIA database (http://gepia.cancer-pku.cn). The data suggest that mRNA expression of USP14 was positively correlated with that of HIF1-α target genes, including MET, VEGF-A, MMP2, and TGFb3 (Fig. 2A), but not TWIST and EPO (Figure S2A, B). We thus supposed that USP14 might be involved in the modulation of HIF1-α-induced transactivation. A luciferase assay was then performed under normoxia and hypoxia conditions to determine the regulation function of USP14 on HIF1-α action. Our results demonstrated that HIF1-α-mediated transcriptional activity was enhanced by USP14 under hypoxia conditions in HCCLM3 cells (Fig. 2B). Furthermore, overexpression of USP14 significantly upregulated the transcription of the endogenous HIF1-α target genes, including MET, VEGF-A, and MMP2 (Fig. 2C). Taken together, our results suggest that USP14 enhances HIF1-α-mediated transactivation.

### USP14 maintains HIF1-α stability via its deubiquitination activity

**Fig. 3:**
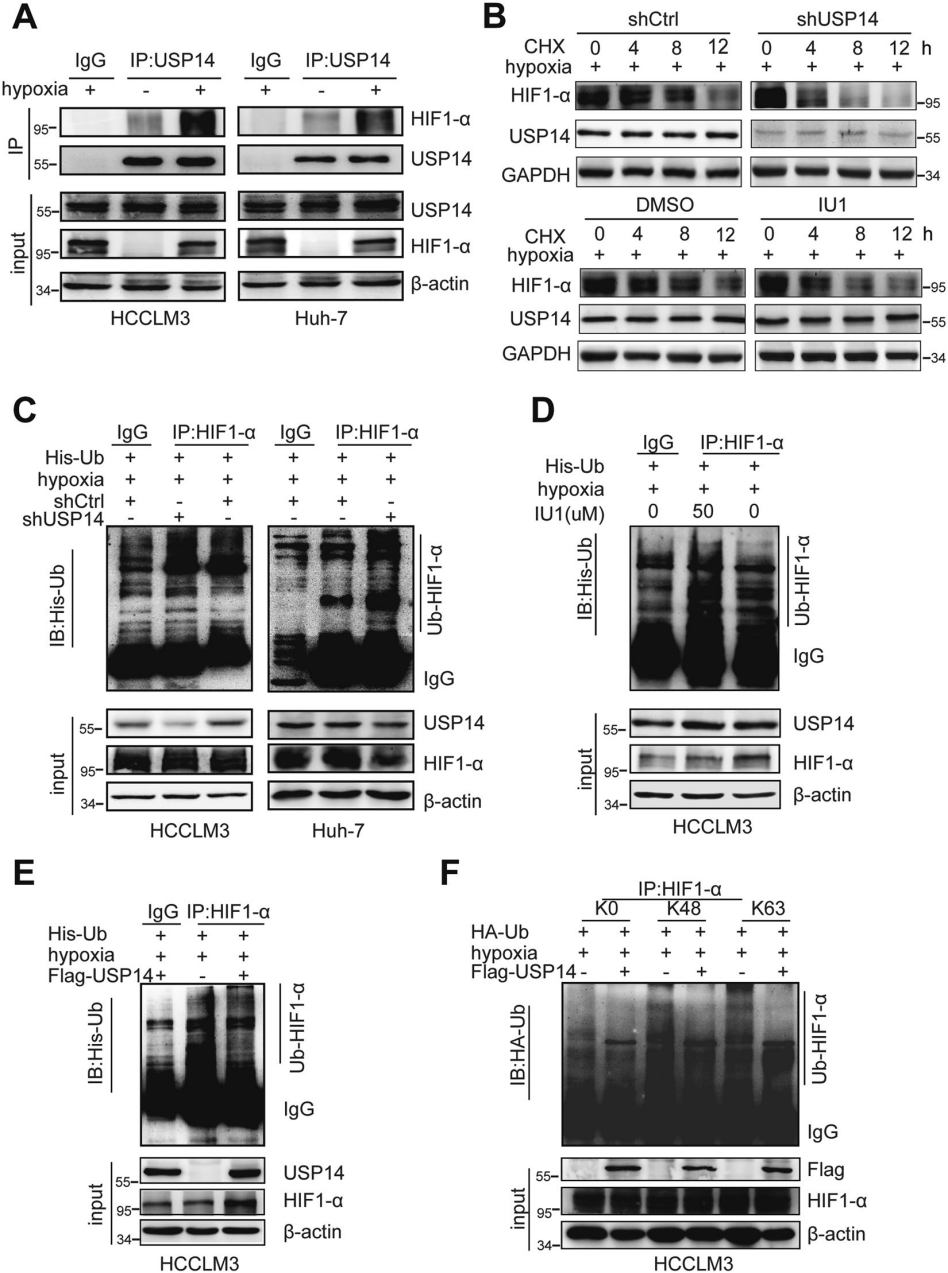
USP14 interacts with HIF1-α and participates in deubiquitination of HIF1-α. **A** Immunoprecipitation experiments showed that endogenous USP14 interacted with HIF1-α in both HCCLM3 and Huh-7 cells. Cells were treated with or without CoCl2 (100 mM). **B** HCCLM3 cells stably knocking down of USP14 or IU1 addition were treated with cycloheximide (CHX) as indicated. Western blot was used to detect HIF1-α and USP14 protein levels. **C** The control and USP14 knockdown HCCLM3 and Huh-7 cells were treated with MG132 at 20 μM for 6 h. The cell lysates were then immunoprecipitated with anti-HIF1-α, and the immunocomplexes were immunoblotted with anti-His. **D** Protein lysates were collected from HCCLM3 cells treated with IU1 for 48 h. A co-immunoprecipitation assay was performed using HIF1-α antibody and immunoblotted with anti-His. Cells were exposed to MG132 (20 μM) for 6 h before harvest. **E** HCCLM3 cells were transfected with Flag-USP14 or empty vector and followed by immunoprecipitation with anti-HIF1-α. **F** HCCLM3 cells with ectopic expression of Flag-USP14 and HA-tagged different ubiquitin mutants, including K0 (lysineless), K48 (only K48-linked-Ub), and K63 (only K63-linked-Ub) were treated with MG132 (20 μM) for 6 h as indicated. The cell lysates were immunoprecipitated by using anti-HIF1-α. The ubiquitination levels of HIF1-α were detected using anti-HA. All the above hypoxic environment refers to hypoxia-induced by CoCl2 at 100 μM.

Having established that USP14 as a deubiquitinase enhances the transcriptional activity of HIF1-α, we thus turned to analyze the mechanism underlying the modulation function of USP14 on HIF1-α action. As shown in Fig. 2D, CoCl2- induced hypoxia significantly increased HIF1-α protein expression compared to the normoxic state. USP14 depletion significantly decreased HIF1-α protein level, but not HIF1-α mRNA level in HCC cells, suggesting that USP14 may be involved in the maintenance of HIF1-α stability (Fig. 2E, F, Figure S2D). Furthermore, the results in co-IP experiments showed that the endogenous USP14 interacted with HIF1-α in HCCLM3 and Huh-7 cells under hypoxia conditions induced by CoCl2 (Fig. 3A).

To further examine the influence of USP14 on HIF1-α stability in hypoxia, HCC cells were treated with protein synthesis inhibitor, cycloheximide (CHX). Knockdown of USP14 or USP14 inhibitor specific for its deubiquitination activity (IU1) [18, 19] accelerated the degradation of HIF1-α protein in HCCLM3 cells with the treatment of CoC12 (Fig. 3B and Figure S3C). Moreover, the ubiquitination assay demonstrated that the ubiquitination level of HIF1-α was significantly increased by USP14 depletion or IU1 treatment in HCCLM3 and Huh-7 cells (Fig. 3C, D). While the ectopic expression of USP14 decreased the ubiquitination level of HIF1-α (Fig. 3E).

Next, we want to determine the specific type of HIF1-α polyubiquitin influenced by USP14. Under hypoxia conditions induced by CoCl2, HCCLM3 cells were separately transfected with Flag-USP14 and different ubiquitins (K0-, K48-, or K63-only ubiquitin-HA tagged). Then, HIF1-α proteins were purified and subjected to western blot analysis using an anti-HA antibody. Our results demonstrated that K48- and K63- linked ubiquitination on HIF1-α were substantially decreased by the overexpression of USP14 (Fig. 3F). Taken together, our results suggest that USP14 participates in the maintenance of HIF1-α stability by its deubiquitinase activity in hypoxia.

### USP14 increases cell proliferation in HCC cell lines

**Fig. 4:**
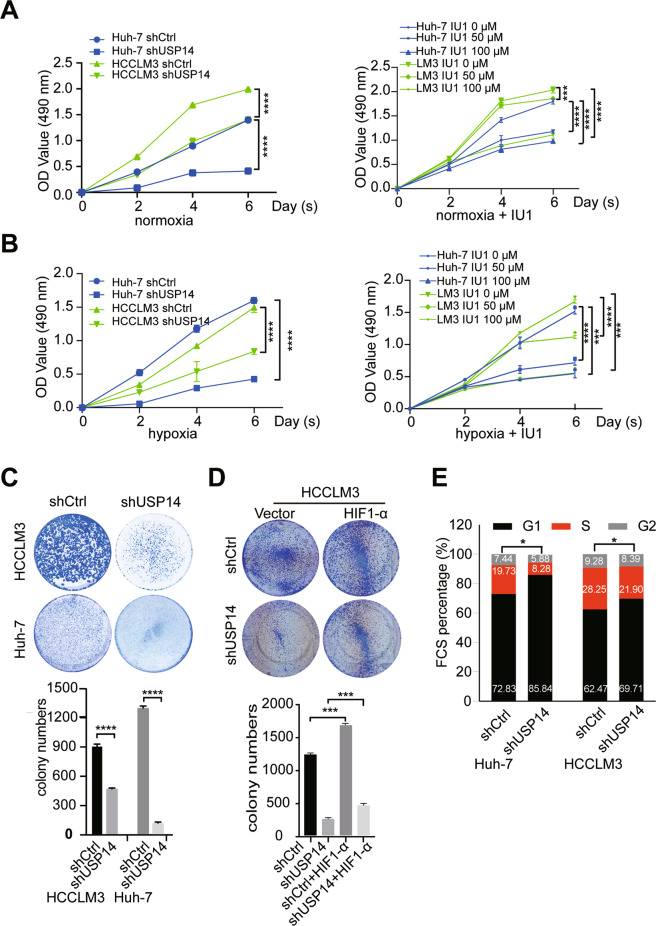
USP14 increases cell proliferation in HCC cells. **A**, **B** The MTS assay was conducted to detect the proliferation of USP14 knockdown and IU1 treated HCC cells in normoxic condition (**A**) and CoCl2 treatment to mimic hypoxia condition (**B**). **C** Colony formation assay indicated the effects of USP14 on cell growth in HCCLM3 and Huh-7 cells. **D** Effects of HIF1-α on the proliferation inhibition induced by USP14 knockdown in HCCLM3 cells as illustrated by colony formation assay. **E** Cell-cycle analysis by flow cytometry for HCC cells with knockdown of USP14 (shUSP14). *P < 0.05; **P < 0.01; ***P < 0.001; ****P < 0.0001; ns = no significance.

To assess the biological function of USP14 in the development and progression of HCC, we firstly examined USP14 protein expression in a panel of immortalized hepatocyte and HCC cell lines. Compared with the immortalized HL-7702 hepatocyte cell line, USP14 is significantly higher in Huh-7 cells and moderately higher in PLC/PRF/5, HCCLM3, and BEL-7402 cells, but weakly expressed in SMMC-7721 and Hep G2 cells (Figure S3A). We performed a knockdown of USP14 expression in Huh-7 and HCCLM3 cells using shRNA and verified USP14 knockdown efficiency by western blot (Figure S3B). Growth curves analysis showed that USP14 depletion and its inhibitor IU1 inhibited cell proliferation under normoxia and hypoxia conditions (Fig. 4A, B). Moreover, compared with control, 50 μM IU1 administration significantly reduced cell viability in time-dependent manners (Fig. 4A, B).

Consistently, colony formation experiments demonstrated that knockdown of USP14 formed a lower number of colonies than that of control (Fig. 4C). Additionally, in USP14-silenced HCC HCCLM3 cells, the expression plasmid of HIF1-α was transfected for rescue experiments. The restoration of HIF1-α expression partially attenuated the suppression of the proliferative ability induced by USP14 depletion (Fig. 4D). Finally, we then performed flow cytometry analysis to examine the effect of USP14 on cell-cycle distribution. The results revealed that knockdown of USP14 induced an increase in the percentage of G1 phase cells as well as a decrease in the percentage of G2/M phase cells (Fig. 4E). The above results suggested USP14 promotes HCC cell proliferation partially in a HIF1-α-dependent manner.

### USP14 promotes cell migration, invasion, and VM formation in HCC cells

**Fig. 5:**
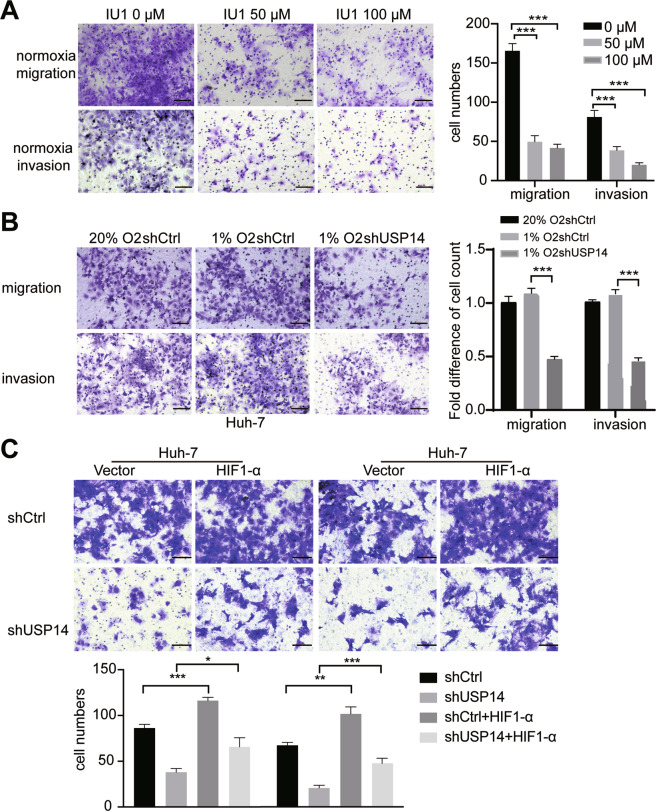
USP14 promotes cell migration and invasion in HCC cells. **A** The migration and invasion ability of HCC cells were inhibited by 1U1 at a dose-dependent manner under normoxic conditions. Scale bar, 100 μM. Quantification of the numbers of migration and invasion cells is shown as histograms on the right. In the histogram, the bars represent the mean ± SD (*n* = 3), *P < 0.05, **P < 0.01, ***P < 0.001. **B** USP14 depletion decreases the migration and invasion ability of HCC cells in hypoxia. Hypoxia groups were incubated in 1%O_2_/5%CO_2_/balance N_2_. Scale bar, 100 μM. Data are shown as mean ± SD (*n* = 3), *P < 0.05, **P < 0.01, ***P < 0.001. **C** HIF1-α attenuated the migration and invasion inhibition induced by USP14 knockdown in Huh-7 cells. Scale bar, 100 μM. *P < 0.05; **P < 0.01; ***P < 0.001; ****P < 0.0001; ns = no significance.

Hypoxia is a common phenomenon in HCC due to the large consumption of oxygen and energy, which affects the metastasis, angiogenesis, and survival of cells [20]. In the hypoxic condition, HIF-1α protein is accumulated and induces the activity of its downstream vascular endothelial growth factor (VEGF) genes, thereby promoting VM formation [21]. HIF-1α was reported to induce vasculogenic mimicry formation in several human cancer cells [22, 23]. We thus turned to examine the effects of USP14 on migratory and invasive behaviors of HCC cells. Firstly, cell migration and invasion in Huh-7 cells were markedly inhibited under normoxia conditions by IU1 supplementation at concentrations of 50 and 100 μM (Fig. 5A). Transwell assay demonstrated that knockdown of USP14 showed a similar effect both in normoxia and hypoxia (Fig. 5B). Subsequent rescue assay showed that the inhibition was partially reversed by HIF1-α (Fig. 5C).

**Fig. 6:**
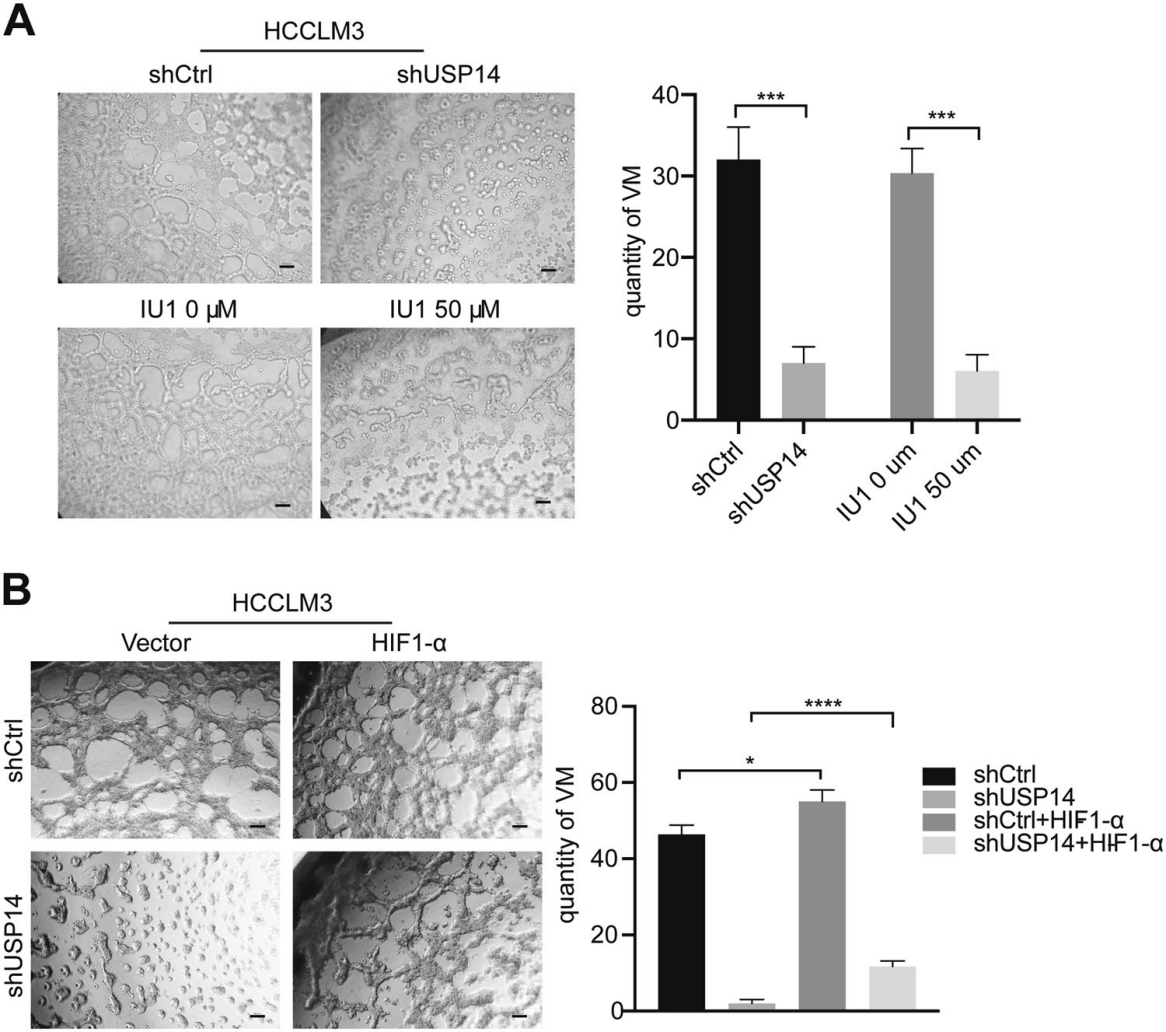
USP14 promotes VM formation in cells*.* **A** VM formation of the HCC cells were extremely restricted by both shUSP14 and IU1. Scale bar, 100 μM. **B** HIF1-α eliminated the vasculogenic mimicry restraint induced by USP14 knockdown in HCCLM3 cells. Scale bar, 100 μM. *P < 0.05; **P < 0.01; ***P < 0.001; ****P < 0.0001; ns = no significance.

Vasculogenic mimicry (VM), an abnormal blood supply pattern, is associated with poor patient prognosis and is considered as a loophole for antiangiogenetic therapy and is critical for tumor growth and metastasis [24]. To investigate the function of USP14 in VM, we used a Matrigel-based tube formation assay. As shown in Fig. 6A, knockdown of USP14 and administration with 50 μM IU1 remarkably inhibited tube formation. Moreover, the suppression of VM induced by USP14 depletion was partially attenuated by the restoration of HIF1-α expression (Fig. 6B). These findings indicated that USP14 serves upstream of HIF1-α to regulate HCC progression.

### USP14 depletion or IU1 treatment suppresses HCC cell growth in mice

**Fig. 7:**
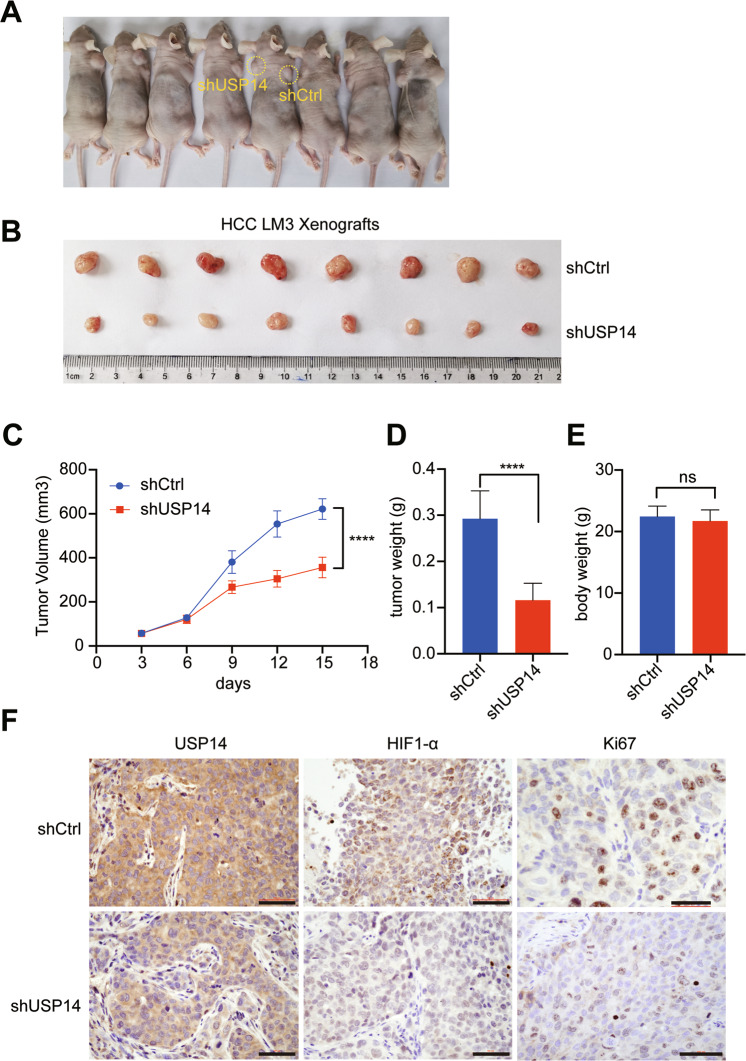
USP14 depletion suppresses HCC cell growth in mice. **A** BALB/c nude mice were subcutaneously injected with HCC HCCLM3 cells stably expressing either human USP14-specific shRNA (left) or control shRNA (right). Representative tumor xenograft images (**B**), tumor volume (**C**), tumor weighed (**D**), and mouse body weight (**E**) were shown ***P < 0.001; ****P < 0.0001; ns = no significance. **F** Immunohistochemistry staining for USP14, HIF1-α, and Ki67. Scale bar, 100 μM.

To further confirm the proliferation potential of USP14, the control and USP14-silenced HCCLM3 cells were subcutaneously injected into nude mice (Fig. 7A, B). The size of xenograft tumors from USP14 stable knockdown cells were much smaller than those formed by the control cells (Fig. 7C). Additionally, the tumor volume from shUSP14 cells demonstrated a lower growth rate than tumors from the shCtrl cells (Fig. 7D). Moreover, the tumor weight of the shUSP14 group was markedly lower than that of the control group (Fig. 7E). However, no obvious body weight loss happened in the two groups (Fig. 7F). Subsequently, the level of USP14, Ki67, and HIF1-α were evaluated using immunochemical staining. We found that the expression of USP14, Ki67, and HIF1-α, which promote proliferation, were decreased in the shUSP14 group (Fig. 7G). Collectively, these data demonstrated that USP14 promoted HCC proliferation and tumor growth.

**Fig. 8:**
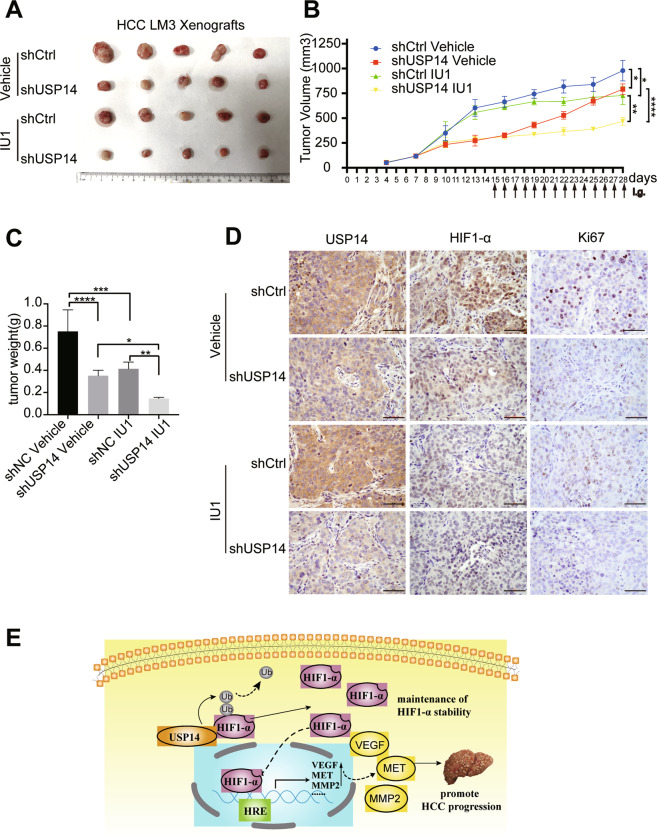
Co-treatment of IU1 and USP14 knockdown suppresses HCC cell growth in mice. **A**–**C** BALB/c nude mice, at two weeks after inoculation with HCCLM3 cells stably expressing either human USP14-specific shRNA or control shRNA, were administrated orally with IU1 (40 mg/kg, every two days) for 14 days. Xenograft images (**A**), tumor volume (**B**), and tumor weight (**C**) were presented. *P < 0.05; **P < 0.01; ***P < 0.001; ****P < 0.0001; ns = no significance. **D** Representative micrographs of immunohistochemistry staining for USP14, HIF1-α, and Ki67 in explanted tumor tissues. Scale bar, 100 μM. **E** The schematic diagram of USP14 promotes the procession of HCC by stabilizing HIF1-α and activating HIF1-α transcription via deubiquitination.

To further verify whether ablation of USP14 or/and a selective deubiquitination activity inhibitor of USP14 (IU1) could restrain HCC growth in animals, we implanted subcutaneously USP14-depleted HCCLM3 cells stably expressing USP14-specific shRNA (shUSP14) or control shRNA (shCtrl) and monitored tumor growth as they were treated with IU1 or vehicle (Figure S4A, B). Importantly, a synergic effect of oral administration of IU1 in combination with lentivirus-mediated USP14 knockdown on tumor growth inhibition was observed upon co-treatment of the two different ways to inhibit USP14 for 14 days (Fig. 8A). Tumor volumes and tumor weight exerted remarkably declined in the combination therapy as compared with the lentivirus-mediated USP14 knockdown or IU1 alone treatment (Fig. 8B, C). However, no obvious body weight loss was observed in each experimental group (Figure S4C). Furthermore, immunohistochemical experiments demonstrated that the protein levels of USP14, HIF1-α, and Ki67 were decreased in the IU1/ shUSP14 combined group compared with the signal treatment (Fig. 8D).

## Discussions

It has been widely accepted that solid cancers are characterized by their limited oxygenation (called hypoxia) [25]. As an important metabolic organ, the local partial pressure of oxygen in the liver can even be as low as 0.8% [26]. HIF1-α, a crucial regulator of oxygen homeostasis [27], is associated with metastasis [28], angiogenesis [29], and sorafenib resistance [30] in HCC. USP14 has been recognized as a key regulator of proliferation [31], apoptosis [32] in tumor [33]. However, the downstream molecules of USP14 are still poorly understood. In this study, our results have demonstrated that USP14 interacts with HIF1-α, and co-activates HIF1-α-mediated transactivation. USP14 promotes tumor progress in HCC even under hypoxia conditions. We further provided the evidence to show the molecular mechanism underlying the function of USP14 on maintenance of HIF1-α stability and enhancement of HIF1-α-induced transcriptional activity via its deubiquitination activity in HCC (Fig. 8E).

Previous researches reported an obvious negative correlation between USP14 expression and survival time of patients with non-small-cell lung cancer [34]. Consistent with the previous findings of USP14 in hepatocellular carcinoma [35], our study has demonstrated the key role of USP14 in determining the clinical severity and prognosis of patients, and thus to explore the anticancer efficacy of USP14 inhibitor IU1 as a potential hepatocellular carcinoma therapeutic agent in vivo and in vitro. USP14 acts as a deubiquitinating enzyme to avoid the degradation of substrate proteins. In normal liver tissues (normoxia dominated), the USP14 protein level is lower than that of tumor tissues, and HIF1-α is lower expressed. When hepatocellular carcinoma occurs, the USP14 protein level is abnormally increased in HCC tissues. With the rapid growth of HCC, the tumor cells would respond to the adverse effects of hypoxia, HIF1-α protein level dramatically increases, at this time, highly expressed USP14 would be involved in the maintenance of HIF1-α protein stability. The accumulation of excess HIF1-α protein enhances the transcription of downstream target genes, which in turn enhances the malignant biological behaviors such as proliferation, invasion and metastasis, and neovascularization of hepatocellular carcinoma cells, leading to tumor progression. Excitingly, we revealed a novel mechanism by which hepatocellular carcinoma tissues tolerate hypoxic adversities and maintain sustained malignant biological behavior through the modulation function of USP14 on HIF1-α-mediated transactivation. Moreover, it has been reported that the expression of HIF1-α is positively correlated with that of HIF1-α target genes, including SNAIL and TWIST, which are involved in tumor metastasis in HCC [36]. Our results demonstrated that the higher protein level of USP14 was not only positively correlated with that of the HIF1-α, but also closely related to the clinical stage, tumor differentiation, and patient prognosis of HCC. These results imply a rational basis for targeting the USP14-HIF1-α axis to control HCC progression.

Deubiquitinase is usually considered that it can remove ubiquitin marks from substrates to restrain the substrates from degradation [37]. Among the known ubiquitin linkage-type, including K6, K11, K27, K29, K33, K48, and K63 [38], K48 is well known for tagging protein substrates for proteasomal degradation [39], while K63 has been reported to play the roles in both transcriptional regulation [40] and protein degradation [41]. In the present study, we provided the evidence that both K48- and K63-linked polyubiquitination on HIF1-α were significantly reduced by overexpression of USP14, suggesting that USP14 is involved in the maintenance of HIF1-α stability by triggering K48- and K63-linked deubiquitination on HIF1-α via its deubiquitination activity. On the other hand, our results also indicate that USP14 may modulate HIF1-α action through K63-linked deubiquitination beyond protein degradation. It would be fantastic to find other biological functions for K63-linked deubiquitination on HIF1-α by USP14 in future work.

Traditionally, the rapid breakdown (proteolysis) of HIF1-α protein is closely related to the microenvironmental oxygen content. Recently, increasing evidence suggests that HIF1-α levels accumulate even in normoxia, when tumor suppressor genes such as Phosphatase and Tensin homolog (PTEN) or p53 are suppressed or oncogenes such as RAS and phosphoinositide 3-kinase (PI3K) are activated [42]. The results in this study suggested that USP14 is upregulated and plays a carcinogenic role in HCC, including promoting proliferation, invasion, and metastasis. Therefore, the regulatory function of USP14 on HIF1-α action revealed under hypoxia conditions in this work may also be applicable for the normoxic state in tumor cells.

Sorafenib is a multikinase inhibitor that blocks tumor cell proliferation by inhibiting serine/threonine kinase isoforms of Raf, Raf-1, and B-Raf, leading to the inhibition of mitogen-activated protein kinase/extracellular signal-regulated kinase (ERK) signaling pathways, decreased expression of cyclin D1 and cell cycle arrest. As a first-line drug approved for therapy in patients with advanced liver cancer by the FDA, sorafenib was once widely used for HCC therapy, however, the effect of sorafenib on the treatment of HCC patients is still unsatisfactory [43]. The possible mechanism for the resistance of sorafenib treatment has been considered that inhibition of tumor angiogenesis by blocking VEGF signal further exacerbates hypoxia within the tumor [44]. Our results showed that USP14 was involved in the upregulation of HIF1-α-induced the expression of *VEGF*, *MMP2*, or *MET*. Additionally, USP14 depletion and its inhibitor IU1 significantly inhibited cell proliferation, migration, and angiogenesis in HCC, suggesting that USP14 might be a potential therapeutic target for HCC. Thus, the combined application of sorafenib and USP14 inhibitors will hopefully enhance the sensitivity of sorafenib. However, before targeting USP14 strategy applied to clinical practice, the following issues need to be carefully considered and further explored: 1) does USP14 have an extra effect on other HIF family members HIF1-β; 2) what potential side effects following the systemic administration of USP14 inhibitors IU1, especially in the cardio, cerebral, renal and reproductive system. A reasonable solution to the above issues will facilitate improving the therapeutic effect of USP14 inhibitor in HCC in the future.

Taken together, our findings suggest a novel mechanism and functional link between USP14 and HIF1-α in the malignant biological behavior of HCC, providing a novel therapeutic target for HCC therapy.

## Materials and methods

### Chemical reagents and plasmid constructs

IU1 (S7134) was obtained from Selleckchem (Houston, USA). Cycloheximide (CHX) was purchased from Sigma-Aldrich and MG132 was purchased from Abmole. Dimethylsulphoxide (DMSO) was used to dissolve these agents and stored at −20 °C.

To construct the USP14 expression plasmid, the cDNA encoding the human *USP14* gene was amplified and inserted into the PcDNA3.1 plasmid with Flag tag at its N-terminus. The Luciferase reporter plasmid containing hypoxia response elements (HRE) was kindly provided by Navdeep Chandel (Addgene plasmid # 26731; http://n2t.net/addgene:26731; RRID: Addgene_26731). HA-tagged HIF1-α expression plasmid was purchased from Sion Biological Co., Ltd.

### Cell culture

Human HCC cell lines (BEL-7402, HCCLM3, Huh-7, PLC/PRF/5, HepG2, and SMMC-7721) and normal liver cells HL-7702 were purchased from the Cell Resource Center, Chinese Academy of Science Committee (Shanghai, China). HEK293 cells were kindly provided by Professor Yujie Sun (Nanjing Medical University). The above cell lines were authenticated by STR profiling. Cells were cultured in an appropriate medium supplemented with 10% heat-inactivated fetal bovine serum (FBS, Clark, USA), 100 mg/mL penicillin G, and 100 μg/mL streptomycin at 37 °C in a humidified atmosphere containing 5% CO_2_. Hypoxia condition was performed in a bis-gas incubator with 1%O_2_/5%CO_2_/balance N_2_. We also used cobalt chloride (CoCl_2_, Sigma-Aldrich, USA) to mimic hypoxic conditions. Cells were seeded in dishes or plates and cultured for 24 h, then the medium was removed, and cells were washed with PBS. Afterwards, the cells were treated with 50 μM or 100 μM CoCl_2_ and incubated for 24 or 48 h.

### Lentivirus transfection

Lentiviruses (hU6-MCS-CMV-Puromycin) expressing control shRNA or human USP14-specific shRNA (NM-005151, target sequence: CGCAGAGTTGAAATAATGGAA) were purchased from GeneChem (Shanghai, China). Cells were plated into 3.5 cm dishes. After 24 h, HitransG virus infection reagent (GeneChem, Shanghai, China) and lentivirus mixtures were dissolved in a medium and added into each well. When cells were incubated overnight, the supernatant was replaced with fresh medium and cultured for 48 h. In order to select stably-transfected cells, puromycin (Santa Cruz, CA, USA) was used at the concentration of 2 μg/ml to perform the selection.

### RNA isolation and real time quantitative PCR (qPCR)

Total RNA was extracted from control or shUSP14-transfected HCC cells using Trizol reagent (Invitrogen, Grand Island, NY, USA) and was synthesized via SYBR Real-time PCR Mix Kit (ChamQ^TM^ Universal Vazyme, Nuoweizan Biotechnology Co., Ltd. Nanjing, China). qPCR was performed with a 20 μl reaction mixture containing 0.4 μM forward and reverse primers as protocol instruction. Primers are listed in Supplementary Table S2. SYBR Green fluorescence intensity was determined using a LightCycler^®^ 96 System (Roche Life Science), and the relative expression of target genes was presented as –ΔCT value.

### Western blotting and co-immunoprecipitation (Co-IP)

Cells were lysed in TNE lysis buffer. After quantification using a BCA protein assay kit (Thermo), protein samples were separated by sodium dodecyl sulfate-polyacrylamide gel electrophoresis (SDS-PAGE). These were then transferred onto polyvinylidene difluoride (PVDF) membranes (Millipore). Membranes were blocked in 5% non-fat milk (Bio-Rad) for 1 h at room temperature and then incubated with anti-USP14 (1:1000, sc-398009, Santa Cruz Biotechnology), anti-β-actin (1:500, sc-47778, Santa Cruz Biotechnology), or anti-GAPDH (1:5000, AC002, ABclonal) antibody, followed by incubation with anti-rabbit or anti-mouse secondary antibodies conjugated to horseradish peroxidase (1:50000, IH-001/IH-0031, Jackson ImmunoResearch Laboratories.INC). Immunoreactive proteins were subsequently visualized using the enhanced chemiluminescence (ECL) detection system (Millipore).

Cells were seeded into 10 cm plates and transfected with indicated plasmids. Total proteins were extracted in co-immunoprecipitation (co-IP) lysis buffer (TNE plus Protease inhibitor (PI), Roche Ltd. Switzerland). The cell lysates were incubated with anti-flag(GNI4110-FG, GNI Japan), anti-His (66005-1-Ig, Proteintech) or anti-HA (GNI4110-HA, GNI Japan) antibody or 5 mg anti-USP14 (A300-919A, Bethyl) or anti-HIF1-α (20960-1-AP, Proteintech) or negative control IgG (sc-2027/sc-2025, Santa Cruz Biotechnology) at 4 °C overnight, followed by conjugation with protein G Sepharose^TM^ (GE Healthcare, Sweden) for an additional 4 h. The beads were then washed thrice with lysis buffer and the immunoprecipitants were collected for western blot analysis.

### Ubiquitination assay

Indicated plasmids were transiently transfected into HCCLM3 or Huh-7 cells for 48 h. After incubation with 10 μM MG132 for 6 h, the cells were harvested and lysed in the denature lysis buffer. The His/HA-ubiquitinated HIF1-α protein was purified and immunoblotted with anti-His or anti-HA antibodies. HA-tagged different ubiquitin mutants, including K0 (lysineless), K48 (only K48-linked-Ub), and K63 (only K63-linked-Ub) were used in the study as indicated.

### Luciferase assay

HCCLM3 cells were co-transfected with Flag-USP14, HA-HIF1-α or the negative control plasmids, and pGL-HRE-AdML reporter plasmid by using HiGene (Applygen Technologies Inc). pRL-tkvector was used as the internal control. After 24 h, the cells were assayed using the Dual-Luciferase Reporter Assay System (Promega).

### Cell proliferation, FACS analysis, and three-dimensional culture

MTS assay (CellTiter 96Aqueous One Solution reagent, Promega, USA) was employed to determine the number of viable cells over 6 days. In brief, cells were plated at a density of 3 × 10^3^ cells per well in 96-well plates. MTS reagent was added to each well of the plate at different time points, and the plates were incubated for 4 h in an incubator, and absorbance was measured at 490 nm. Each cell group was plated in three duplicate wells. For colony formation assay, 5 × 10^3^ cells were maintained in medium 10%FBS supplemented for 14 days. Cell cultures were then fixed with methanol and stained with Coomassie blue dye.

For the FACS assay, the cells were grown in six-well plates for 24 h, then were dissociated with trypsin, resuspended in PBS, and fixed in ice-cold 70% ethanol. Next, the cells were incubated with 50 μg/ml PI, 100 μg/ml RNaseA solution, and 0.2% Triton-X-100 at 37 °C for 1 h. The cell-cycle analysis was performed by a FACS flow cytometer (BDBiosciences C6). The software for analysis cell cycle is Modfit.

For 3D culture, matrigel (BD Biosciences, USA) was used to coat the 96-well plate and polymerized it at 37 °C for 4 h to solidify the Matrigel. 1 × 10^4^ HCC cells (30 μl/well) were cultured in the Matrigel-coated plates for 24 h. The formation of capillary-like structures was observed under phase-contrast microscopy (200× magnification). Each experiment was performed in triplicate.

### Transwell migration and invasion assay

Cell migration was assessed using a 24-well transwell chamber (8.0 μm pore size filter; Corning, Canton, NY). Cells were harvested and resuspended in a serum-free culture medium. The cell suspension (200 μl; 6.0 × 10^4^ cells) was added to each upper chamber, and each lower chamber was filled with a 600 μl 10% serum medium. After a 24 h incubation at 37 °C, chambers were gently washed twice with PBS, methanol fixed and then Crystal Violet Staining Solution (Solarbio, Beijing, China) stained. Cells that had traversed the filter to the lower chamber were counted microscopically (200×) in three different fields per filter. For the invasion assay, the cell suspension was added to Matrigel Matrix (Corning, Canton, NY, USA) pre-coated transwells and incubated for 4 h at 37 °C, and the assay was conducted using the same procedure as the migration assay.

### Nude mouse xenograft growth

BALB/c mice (4–5 week, 18–22 g, male) were purchased from Beijing Vital River Laboratory Animal Technology Co., Ltd. and all animal procedures were approved and compiled with the guidelines of the Institutional Animal Care Committee of China Medical University. The nude mice were housed in a quarantine room for inspection for 2–3 days. Then mice were transferred to barrier facilities in the animal facility of China Medical University. HCCLM3 cells were infected with lentivirus containing USP14 shRNA or control shRNA. Mice were subcutaneously inoculated with 10^6^ viable cells. After 2-weeks, the inoculated mice were randomly divided into 2 groups and orally administered with IU1 (40 mg/kg/d) or vehicle for 14 days. On the 28th day, the mice were sacrificed by cervical dislocation, and primary tumors were excised and weighed. Tumor volumes were calculated and mouse body weight were measured every other day.

### Clinical samples and immunohistochemistry (IHC)

Thirty-four fresh HCC specimens were collected for determining protein levels of USP14 from First Hospital of China Medical University. Tissue microarrays (TMA) from archival formalin-fixed paraffin-embedded (FFPE) resection specimens consisted of 90 pairs of HCC and their adjacent non-tumorous liver tissues were purchased from Outdo Biotech Co. Ltd. None of the patients had received radiotherapy or chemotherapy before surgery. All samples were anonymous. This project was approved by the Institute Research Ethics Committee of the above two institutions. The clinical implication of USP14 was further determined in UALCAN (http://ualcan.path.uab.edu/index.html), an interactive web portal, to perform in-depth analyses of TCGA transcriptome data, and Human Protein Atlas (www.proteinatlas.org) to compare protein expression of USP14 in HCC with normal liver samples. The prognostic value of the USP14 gene in overall survival and relapse-free survival were analyzed by using Kaplan–Meier Plotter (http://kmplot.com/analysis/) with an auto-select best cutoff.

Tissues were fixed with 10% neutral formalin, embedded in paraffin, and 4 μm thick sections were prepared. In brief, immunohistochemistry (IHC) staining was performed as follows. The sections were deparaffinized, hydrated, and soaked in 3% H_2_O_2_ for 15 min at room temperature, and subsequently incubated with anti-USP14 (1:200, sc-398009, Santa Cruz Biotechnology), anti-HIF1-α (1:500, 20960-1-AP, Proteintech) antibodies, and anti-Ki67 (1:200, sc-23900, Santa Cruz Biotechnology) antibodies at 4 °C overnight. The slides were incubated with biotinylated goat anti-rabbit antibodies for 1 h and stained with diaminobenzidine (DAB; Maixin Biotechnology, Fuzhou, China), followed by counterstaining with hematoxylin (Maixin Biotechnology).

IHC staining was performed on an HCC tissue microarray (TMA). Stained sections were evaluated in a blinded manner without prior knowledge of the clinical information using the German immunoreactive score, Immuno- Reactive-Score (IRS). Briefly, the IRS assigns sub-scores for immunoreactive distribution (0–4) and intensity (0–3), then multiplies them to yield the IRS score. The percent positivity was scored as “0” (<5%), “1” (5–25%), “2” (25–50%), “3” (50–75%,), “4” (>75%). The staining intensity was score as “0” (no staining), “1” (weakly stained), “2” (moderately stained), and “3” (strongly stained). The final USP14 expression score was calculated with the value of percent positivity score plus staining intensity score, which ranged from 0–12. We estimated IRS by averaging the values in three fields at ×200 magnification for each specimen. The scores were performed by two independent investigators. The median IHC score of 6.0 was chosen as the cut-off value to define the high and low expression.

### Statistical analysis

The data of all experiments were analyzed by the Statistical Product and Service Solutions (SPSS) (25.0) statistical software program. Data were presented from three independent experiments. The relationship between USP14 expression and clinical features of HCC was assessed by the Chi-squared test. Data described as mean ± SD and Student’s *t*-test or one-way ANOVA were used to determine differences among groups. Survival curves were estimated by the Kaplan–Meier method and compared by a log-rank test. *P*-value less than 0.05 was considered statistically significant.

## Supplementary information

Supplementary Figures-legends

Supplementary Table S1

Supplementary Table S2

Supplementary Table S3

Supplementary-figure 1

Supplementary-figure 2

Supplementary-figure 3

Supplementary-figure 4

## Data Availability

All data generated or analyzed during this study are included in this published article and its supplementary information files. The datasets used and analyzed during the current study are available from the corresponding author on reasonable request.
