# Supplementary material for: USP14 maintains HIF1-α stabilization via its deubiquitination activity in hepatocellular carcinoma
Source: Cell Death Dis. 2021 Aug 21;12(9):803. doi: 10.1038/s41419-021-04089-6 (PMC8380251; doi:10.1038/s41419-021-04089-6)
Supplement: Supplementary file 1 — Supplementary Figures-legends [file 41419_2021_4089_MOESM1_ESM.docx]

**USP14 maintains HIF1-α stabilization via its deubiquitination activity to promote hepatocellular carcinoma progression**

Chi Lv^1, 3^, Shengli Wang^1^, Lin Lin^1^, Chunyu Wang^1^, Kai Zeng^1^, Yiming Meng^2^, Ge Sun^1^, Shan Wei^1^, Yefu Liu^2^, and Yue Zhao^1, 2^*

^1^Department of Cell Biology, Key laboratory of Cell Biology, Ministry of Public Health, and Key laboratory of Medical Cell Biology, Ministry of Education, School of Life Sciences, China Medical University, Shenyang City 110122, Liaoning Province, China

^2^Department of Hepatopancreatobiliary Surgery, Cancer hospital of China Medical University, Liaoning Province Cancer Hospital. No. 44, Xiaoheyan road, Dadong district, Shenyang, Liaoning 110042, China

^3^Anorectal Surgery Ward, Department of General Surgery, Shengjing Hospital of China Medical University, Shenyang City, Liaoning Province, 110004, China

* To whom correspondence should be addressed.

Yue Zhao. Department of Cell Biology, Key laboratory of Cell Biology, Ministry of Public Health, and Key laboratory of Medical Cell Biology, Ministry of Education, School of Life Sciences, China Medical University, No.77 Puhe Road, Shenyang North New Area, Shenyang City 110122, Liaoning Province, China

Tel: +86 24 31939077; Fax: +86 24 31939077; email: yzhao30@cmu.edu.cn

ORCID: https://orcid.org/0000-0001-8983-0024

**SUPPLEMENTARY DATA**

**Supplementary Figure Legends**

**Supplementary Figure S1. Expression of USP14 in HCC and its relationship with clinicopathological features.**

(A) Studies from TCGA dataset presented the increase of USP14 mRNA in HCC samples. (B) Immunohistochemical analysis of USP14 expression in HCC and normal liver tissue. Data derived from the Human Protein Atlas database. bars, 100 μm. (C and D) Box plot showed the correlation between USP14 and HCC stages and histological grades in UALCAN database. (E and F) Survival curves from Kaplan-Meier plot profiles for HCC patients stratified by high and low expression of USP14.

**Supplementary Figure S2. The correlation between HIF1-α’s target gene and USP14 expression.**

(A and B) Studies from the GEPIA database presented the correlation between USP14 mRNA expression and HIF target genes’ mRNA of TWIST and EPO. (C) The effect of ectopic expression of USP14 on the expression of HIF1-α target genes under normoxia condition. (D) The effect of USP14 knockdown on mRNA expression levels of HIF1-α in Huh-7 and LM3 cell lines by qRT-PCR.

**Supplementary Figure S3. USP14 protein expression in different cell lines.**

(A) Various protein expression levels of USP14 in a panel of HCC cell lines. (B) Huh-7 and LM3 cells were infected with lentiviral particles expressing shRNA targeting USP14. The knockdown efficacy was detected by the western blot analysis. (C) HCCLM3 cells stably knocking down of USP14 or IU1 addition were treated with cycloheximide (CHX) as indicated. Western blot was used to detect HIF1-α and USP14 protein level. Quantification of protein expression is shown as histograms. In the histogram, the bars represent the mean ± SD (n = 3), *P < .05, **P < .01, ***P < .001, ns=no significance.

**Supplementary Figure S4. Co-treatment of IU1 and lentivirus-mediated USP14 knockdown suppresses the growth of HCC in vivo.**

(A and B) Representative photograph showing the xenograft tumor from LM3 cells with shCtrl (right) were smaller than shUSP14 cells (left). (C) The difference of mice weight among groups.
