# Supplementary material for: USP14 maintains HIF1-α stabilization via its deubiquitination activity in hepatocellular carcinoma
Source: Cell Death Dis. 2021 Aug 21;12(9):803. doi: 10.1038/s41419-021-04089-6 (PMC8380251; doi:10.1038/s41419-021-04089-6)
Supplement: Supplementary file 2 — Supplementary Table S1 [file 41419_2021_4089_MOESM2_ESM.docx]

| **Supplementary Table S1.**  Correlation of USP14 expression and clinical features of HCC patients | | | | |
| --- | --- | --- | --- | --- |
| Variable | USP14 | | | |
|  | All cases | Low  expression | High  expression | *P* value ^a^ |
| Age(years) ^b^ |  |  |  | 0.428 |
| <54 | 42 | 21(50.0%) | 21(50.0%) |  |
| ≥54 | 48 | 28(58.3%) | 20(41.7%) |  |
| Gender |  |  |  | 0.361 |
| Male | 78 | 41(52.6%) | 37(47.4%) |  |
| Female | 12 | 8(66.7%) | 4(33.3%) |  |
| Cirrhosis |  |  |  | 0.388 |
| Yes | 33 | 16(48.5%) | 17(51.5%) |  |
| No | 57 | 33(57.9%) | 24(42.1%) |  |
| Tumor size (cm) |  |  |  | 0.372 |
| <5 | 35 | 17(48.6%) | 18(51.4%) |  |
| ≥5 | 55 | 32(58.2%) | 23(41.8%) |  |
| Tumor multiplicity |  |  |  | 0.708 |
| Single | 80 | 43(53.8%) | 37(46.3%) |  |
| Multiple | 10 | 6(60.0%) | 4(40.0%) |  |
| Perihepatic organ invasion | |  |  | 0.398 |
| Yes | 86 | 46(53.5%) | 40(46.5%) |  |
| No | 4 | 3(75.0%) | 1(25.0%) |  |
| Differentiation |  |  |  | 0.014 |
| Well | 6 | 6(100%) | 0(0.00%) |  |
| Moderate | 68 | 38(55.9%) | 30(44.1%) |  |
| Poor | 16 | 5(31.3%) | 11(68.8%) |  |
| Vascular invasion |  |  |  | 0.298 |
| Yes | 7 | 5(71.4%) | 2(28.6%) |  |
| No | 83 | 44(53.0%) | 39(47.0%) |  |
| Lymph node metastasis |  |  |  | 0.544 |
| Yes | 84 | 44(52.4%) | 40(47.6%) |  |
| No | 3 | 2(66.7%) | 1(33.3%) |  |
| Distant metastasis |  |  |  | 0.456 |
| Yes | 3 | 1(33.3%) | 2(66.7%) |  |
| No | 84 | 45(53.6%) | 39(46.4%) |  |
| TNM |  |  |  | 0.456 |
| I-II | 43 | 21(48.8%) | 22(51.2%) |  |
| III-IV | 44 | 25(56.8%) | 19(43.2%) |  |
| ^a^ Chi-square test, ^b^ Median age. | |  |  |  |
