# Supplementary material for: USP14 maintains HIF1-α stabilization via its deubiquitination activity in hepatocellular carcinoma
Source: Cell Death Dis. 2021 Aug 21;12(9):803. doi: 10.1038/s41419-021-04089-6 (PMC8380251; doi:10.1038/s41419-021-04089-6)
Supplement: Supplementary file 3 — Supplementary Table S2 [file 41419_2021_4089_MOESM3_ESM.docx]

| **Supplementary Table S2.**  Univariate and multivariate analyses of USP14 expression and overall survival | | | | |
| --- | --- | --- | --- | --- |
| Variables | Univariate analysis | | Multivariate analysis | |
|  | HR (95% CI)  *P value* | | HR (95% CI) *P* value | |
| Overall survival |  |  |  |  |
| Age (< 54 vs. ≥54 years) | 1.522（0.902-2.568） | 0.116 |  |  |
| Gender (female vs. male) | 1.308（0.619-2.765） | 0.482 |  |  |
| Liver cirrhosis (yes vs. no) | 0.684（0.394-1.186） | 0.177 |  |  |
| Tumor size (< 5 vs. ≥5 cm) | 1.189(0.698-2.025) | 0.524 |  |  |
| Tumor multiplicity (single vs. multiple) | 0.767(0.306-1.920) | 0.571 |  |  |
| Vascular invasion (yes vs. no) | 0.913（0.330-2.527） | 0.861 |  |  |
| Tumor differentiation | 1.338(0.818-2.354) | 0.224 |  |  |
| LNM (yes vs. no) | 1.899(0.590-6.112) | 0.282 |  |  |
| Distant metastasis (yes vs. no) | 1.495(0.465-4.806) | 0.499 |  |  |
| T stage | 1.445(1.000-2.089) | 0.050 | 0.646(0.186-2.237) | 0.490 |
| Perihepatic organ invasion (yes vs. no) | 3.096（1.116-8.594） | 0.030 | 4.077(0.984-16.891) | 0.053 |
| TNM Clinical stage | 1.408(1.003-1.975) | 0.048 | 2.146(0.612-7.525) | 0.233 |
| USP14 expression (low vs. high) | 2.011(1.190-3.399) | 0.009 | 2.961(1.591-5.510) | 0.001 |
| *LNM* lymph node metastasis, *HR* hazard ratio, *CI* confidence interval | | | | |
