# Supplementary material for: USP14 maintains HIF1-α stabilization via its deubiquitination activity in hepatocellular carcinoma
Source: Cell Death Dis. 2021 Aug 21;12(9):803. doi: 10.1038/s41419-021-04089-6 (PMC8380251; doi:10.1038/s41419-021-04089-6)
Supplement: Supplementary file 4 — Supplementary Table S3 [file 41419_2021_4089_MOESM4_ESM.docx]

**Supplementary Table S3.**

**Real-time quantitative PCR primers for genes as indicated**

| **Gene** |  | **Sequence5'---3'** |
| --- | --- | --- |
| *β-actin* | F | GTGACGTTGACATCCGTAAAGA |
|  | R | GCCGGACTCATCGTACTCC |
| *GAPDH* | F | GAAGGTGAAGGTCGGACTC |
|  | R | GAAGATGGTGATGGGATTTC |
| *USP14* | F | TGTGCCTGAACTCAAAGATGC |
|  | R | ATATACTGCGCTGAAGCCATTT |
| *HIF1-α* | F | ATCATGTGCTGCTTCGGCTGCAT |
|  | R | AAATTGGACGACCCTCACGGCT |
| *TWIST1* | F | GACTTCCTCTACCAGGTCCTCCAG |
|  | R | TCCAGACCGAGAAGGCGTAGC |
| *MMP2* | F | TGCGGCACCACTGAGGACTAC |
|  | R | GCACCTTCTGAGTTCCCACCAAC |
| *TGFB3* | F | TTCCGCTTCAATGTGTCCTCAGTG |
|  | R | CGATCCTCTGCTCATTCCGCTTAG |
| *MET* | F | GAGGGTCGCTTCATGCAGGTTG |
|  | R | TCACTTCTGGAGACACTGGATGGG |
| *EPO* | F | CACGACGGGCTGTGCTGAAC |
|  | R | ACCTCCATCCTCTTCCAGGCATAG |
| *VEGFA* | F | GCCTTGCCTTGCTGCTCTACC |
|  | R | CTTCGTGATGATTCTGCCCTCCTC |
