# Supplementary figures and images for: USP14 maintains HIF1-α stabilization via its deubiquitination activity in hepatocellular carcinoma
Source: Cell Death Dis. 2021 Aug 21;12(9):803. doi: 10.1038/s41419-021-04089-6 (PMC8380251; doi:10.1038/s41419-021-04089-6)

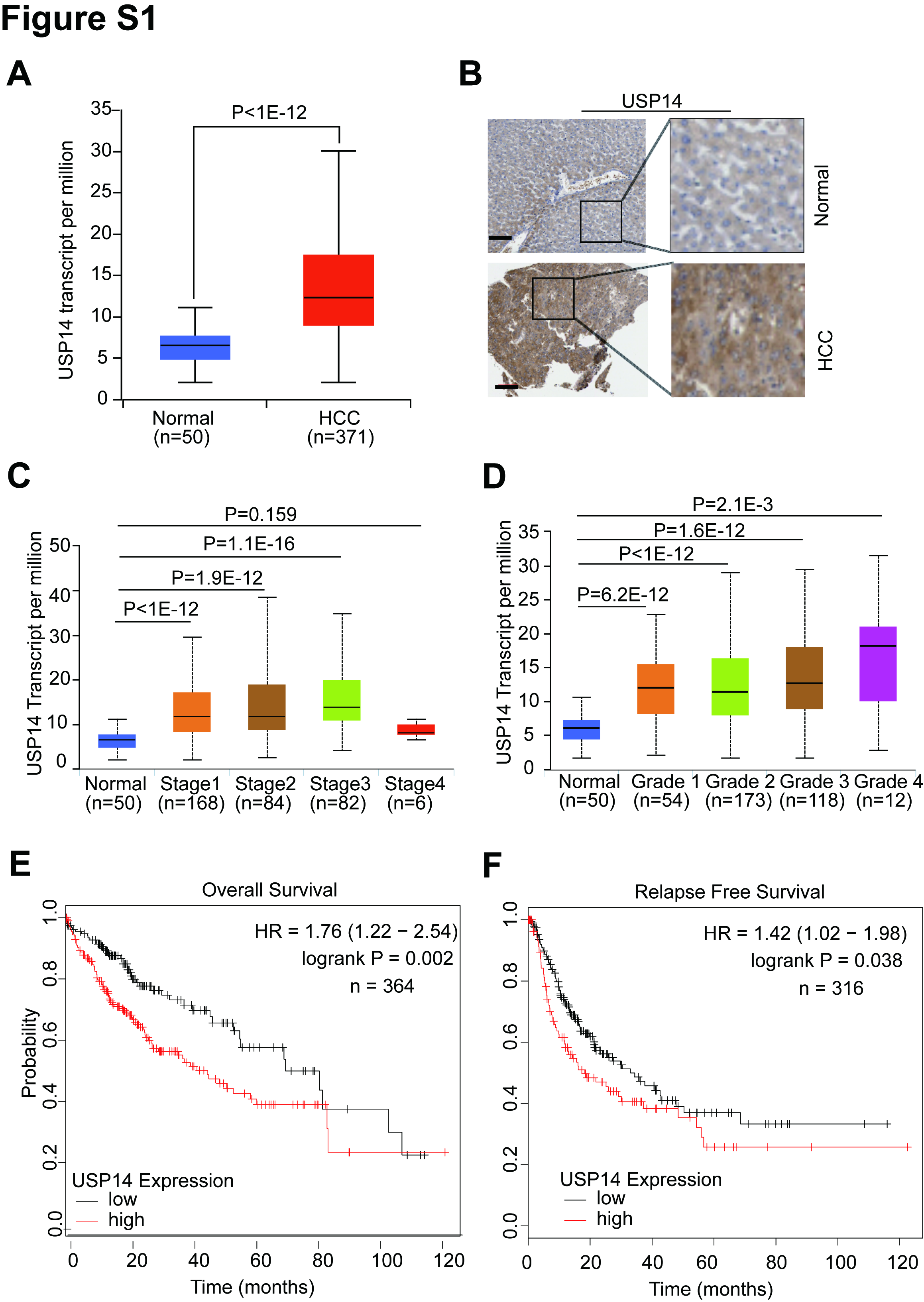

Supplement: Supplementary file 5 — Supplementary-figure 1 [file 41419_2021_4089_MOESM5_ESM.tif]

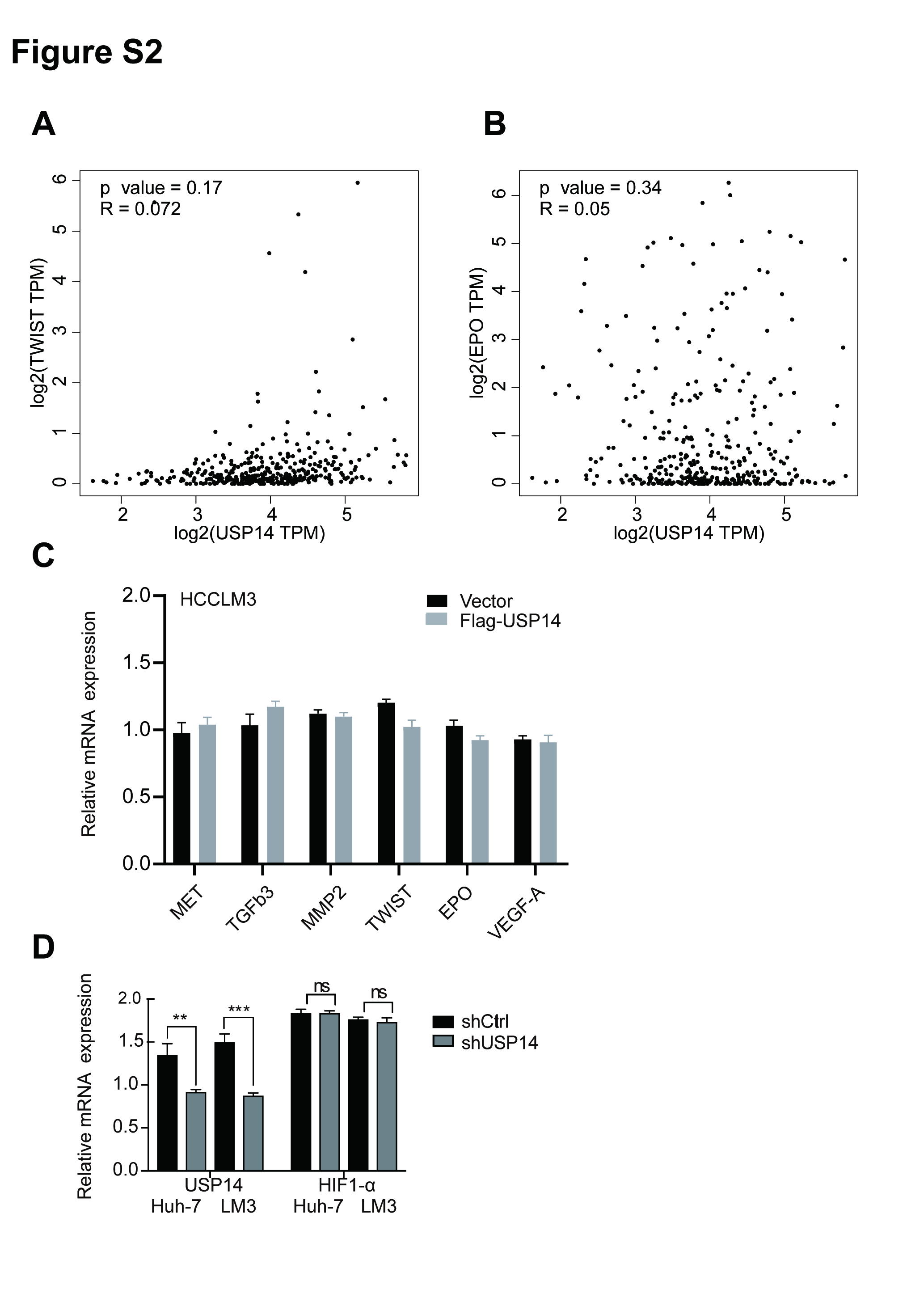

Supplement: Supplementary file 6 — Supplementary-figure 2 [file 41419_2021_4089_MOESM6_ESM.tif]

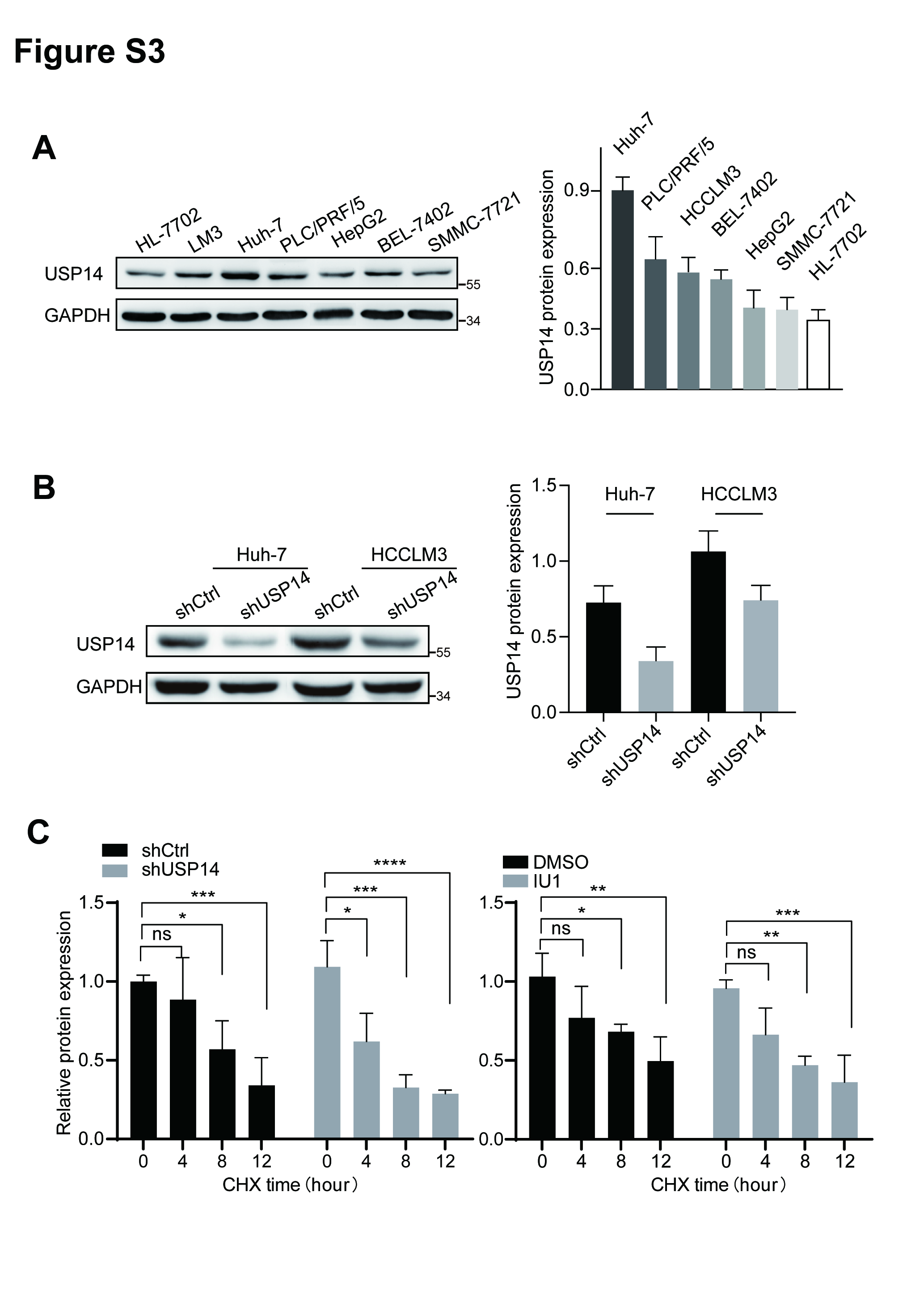

Supplement: Supplementary file 7 — Supplementary-figure 3 [file 41419_2021_4089_MOESM7_ESM.tif]

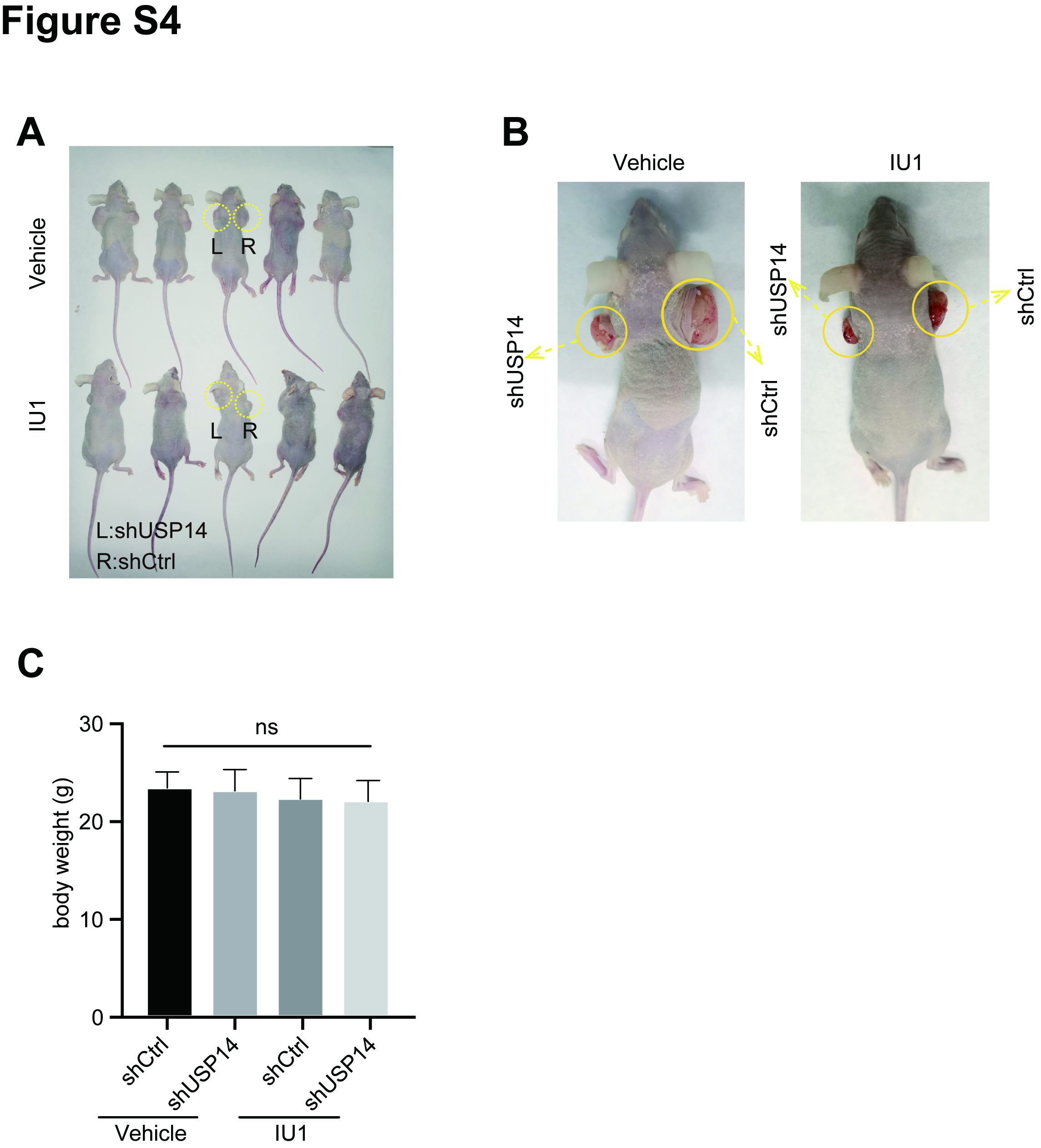

Supplement: Supplementary file 8 — Supplementary-figure 4 [file 41419_2021_4089_MOESM8_ESM.tif]
